# Using gnotobiotic ruminants to dissect host–microbe interactions for sustainable agriculture

**DOI:** 10.3389/fmicb.2026.1771182

**Published:** 2026-04-17

**Authors:** Fernanda Batistel

**Affiliations:** Department of Animal Sciences, Institute of Food and Agricultural Sciences, University of Florida, Gainesville, FL, United States

**Keywords:** gnotobiology, microbial ecology, microbiome, rumen, sustainability

## Abstract

Ruminant animals host one of the most complex gut microbial ecosystems, enabling the conversion of fibrous plant biomass into nutrient-dense foods such as meat and milk, which are essential for global food security. Over time, successive waves of research—from the initial recognition of microbes in the rumen, through anaerobic cultivation, to more recent multi-omics approaches—have progressively expanded our understanding of rumen microbial composition and its links to animal production and greenhouse gas emissions. Despite these advances, most insights into rumen microbial composition and traits of interest are based on associative or correlative evidence, and the host-derived mechanisms that actively shape rumen microbial composition and function remain poorly defined. Early gnotobiotic studies in ruminants demonstrated the value of maintaining animals under defined microbial conditions to dissect host–microbe interactions; however, this experimental capability has largely been lost from contemporary rumen research. This *Perspective* argues that revisiting gnotobiotic ruminant models is both timely and necessary for establishing causal mechanisms that govern host–microbe interactions in the rumen. Integrating gnotobiotic ruminant models is essential for establishing causal relationships between host biology and rumen microbial composition, thereby providing a foundation for biologically informed strategies that can enhance the sustainability of ruminant production systems.

*In science, ideas that were once pioneering are often set aside, only to re-emerge as groundbreaking decades later*.

## 1 Introduction

The gastrointestinal tract represents one of the most intricately co-evolved microbial habitats, yet its structural and functional complexity varies widely among animal hosts. This complexity is greatest in species with large, compartmentalized digestive tracts that consume fiber-rich diets and rely heavily on microbial fermentation to meet their nutritional needs (Muegge et al., 2011). Within this group, ruminants—including, but not limited to, cattle, sheep, and goats—harbor what is arguably the most sophisticated gut microbial ecosystem. The rumen, their specialized first stomach compartment, is estimated to harbor an enormous diversity of microbes, including more 4,800 bacterial species (Stewart et al., 2019), nearly 300 protozoal species (Willians and Coleman, 1992), over 350 fungal species (Wang et al., 2023b), hundreds of archaeal species (Mi et al., 2024), and thousands of viral types (Yan et al., 2023). Through microbial fermentation, fibrous carbohydrates are converted into short-chain fatty acids that provide up to 70% of the animal's energy requirements (Bergman, 1990). Microbial biomass flowing into the lower gastrointestinal tract supplies a large proportion of the host's amino acid requirements (Storm et al., 1983), while the synthesis of B and K vitamins further supports host nutrition (NASEM, 2021). This remarkable metabolic adaptability underpins the ability of ruminants to thrive in diverse environments and likely played a key role in their early domestication.

Historically, ruminants provided not only food but also labor, leather, wool, and fertilizer, shaping the development of human societies (Smith, 1998). Although their roles in labor and textiles have diminished, ruminants remain indispensable to modern food systems. Their unique capacity to convert plant biomass into nutrient-dense foods, such as milk and meat, continues to make substantial contributions to human diets. Beyond calories, these products supply high-quality protein, iron, zinc, vitamin B12, and health-promoting fatty acids such as conjugated linoleic acid. As a result, ruminant-derived foods play a critical role in global nutrition and food security, particularly for vulnerable populations (Adesogan et al., 2020).

As the global population continues to rise (United Nations, 2024), the need to produce ruminant-derived foods sustainably and efficiently is becoming increasingly critical. Although improvements in genetics, nutrition, and management have boosted productivity per animal over the past decades, these gains alone are unlikely to meet future demand. Furthermore, increasing the number of animals is feasible only in regions with underutilized land, but it is not a sustainable option in most countries due to limitations in land and resources, as well as concerns over greenhouse gas emissions from enteric fermentation. Instead, efforts must focus on improving production efficiency by producing more ruminant products with fewer resources while simultaneously reducing environmental impact. Central to this challenge is the microbial ecosystem of the rumen, which is responsible for driving feed conversion and nutrient utilization, but it also contributes to greenhouse gas production.

Despite remarkable advances in our understanding of the rumen microbial ecosystem, key questions remain unresolved. Therefore, this *Perspective* aims to contextualize modern rumen microbial ecosystem research within its historical development and to demonstrate that the field's current reliance on associative and correlative frameworks stems from the loss of experimental systems capable of testing causality. Reintroducing gnotobiotic ruminant models is proposed as a logical and timely approach to address this gap.

## 2 The roads taken: milestones that transformed our understanding of the rumen microbial ecosystem

In 1685, the Swiss anatomist and physician Johann C. Peyer published one of the first systematic explanations of ruminant digestion. In addition to describing the structure of the ruminant stomach compartments and the process of rumination, he characterized digestion as a form of “putrefaction” or “chemical dissolution” (Peyer, 1685), reflecting the 17th-century pre-microbial view of feed degradation in the rumen. Subsequent studies observed that plant material in the rumen generated volatile compounds (Tiedemann and Gmelin, 1831; Sprengel, 1832), as well as the presence of microscopic “animalcules” —later identified as protozoa—in the rumen (Gruby and Delafond, 1843). However, it was only after the pioneering work of Louis Pasteur on the role of bacteria in food fermentation (Pasteur, 1857, 1860a) that microbial activity was hypothesized to be the underlying mechanism of carbohydrate degradation and formation of short-chain fatty acids and gases in the rumen (Zuntz, 1879). Soon after, it was proposed that these short-chain fatty acids were not merely end-products of fermentation but were absorbed through the rumen wall and utilized by the host as an energy source (Tappeiner, 1884). Although initially met with skepticism, the “fermentation hypothesis” inspired decades of research that confirmed its legitimacy. At the same period, researchers also began to explore another important question—whether life could exist in the absence of microbes (Pasteur, 1860b). This pursuit led to the generation of germ-free guinea pigs (Nuttall and Thierfelder, 1896) and, soon after, goats (Küster, 1912). These achievements marked the beginning of experimental gnotobiology—the study of organisms with a known microbial status, including germ-free or selectively colonized animals—providing an unprecedented means to critically investigate the interface between the host and its microbes.

By the mid-20th century, the nutritional importance of microbial fermentation products for the host was well established, but characterization of rumen microbial species remained limited because most are strict anaerobes and could not be cultured using conventional aerobic microbiological techniques. This barrier was overcome by the pioneering work of Robert E. Hungate, who developed a strict anaerobic cultivation technique (Hungate, 1950). This method, along with its subsequent refinements (Bryant, 1972), enabled the isolation and characterization of several rumen microbes, laying the foundation for modern rumen microbiology. During this period, viruses (Hoogenraad et al., 1967) and fungi (Orpin, 1975) were also identified, expanding the known rumen microbial ecosystem. The adoption of pure culture-based approaches marked a decisive shift from descriptive studies of microbial composition to experimental investigations that clarified the physiological roles and metabolic interactions of rumen microbes. For the first time, researchers could associate specific microbes with defined biochemical functions and systematically examine their substrate preferences, growth requirements, metabolic activities, and the interconnected metabolic cascades underlying microbial interactions. Seminal contributions from Marvin P. Bryant (Bryant, 1974; Chung, 1997), James B. Russell (Russell and Rychlik, 2001; Weimer et al., 2010a), Burk A. Dehority (Dehority, 2003; Firkins et al., 2020), and Colin G. Orpin (Orpin, 1984), among others, were central to advancing these research topics. The taxonomic classification of rumen microbes and assessments of microbial community dynamics in response to age, diet, and host factors were based primarily on visual identification and cell counts performed using microscopy. Methanogens were also still classified as “methanogenic bacteria”; they would not receive their current designation as archaea until later (Woese et al., 1990). The development of *in vitro* rumen models, such as dual-flow continuous-culture fermenters (Hoover et al., 1976a,b) and the long-term rumen simulation technique (aka Rusitec; Czerkawski and Breckenridge, 1979a,b) started to provide controlled platforms enabling standardized studies of rumen microbial fermentation. Notably, it was also during the second half of the 20th century that the majority of gnotobiotic research in ruminant animals was conducted. During this time, gnotobiotic techniques and specialized equipment were refined (e.g., Alexander et al., 1973a,b; Cushnie et al., 1981). The capacity to isolate and culture microbial strains responsible for key rumen functions, together with the use of gnotobiotic hosts, enabled studies examining how individual microbes and defined consortia colonize the rumen, interact, and contribute to host digestion, fermentation patterns, and nutrient supply (e.g., Lysons et al., 1971; Stewart and Lysons, 1975; Hobson et al., 1981; Fonty et al., 1992). Although early experiments were often constrained by limited replication and statistical constraints of the time, they nonetheless provided early insights into rumen microbial activity and host–microbe relationships.

Early culture-independent investigations of the rumen microbial ecosystem in the late 1990s were made possible by the foundational work of Carl Woese and George Fox, who demonstrated that conserved ribosomal RNA gene sequences could serve as universal molecular markers for microbial identification (Fox et al., 1977; Woese and Fox, 1977). This framework led to molecular approaches targeting ribosomal RNA genes across different microbial domains—such as the 16S rRNA gene for bacteria and archaea, the 18S rRNA gene for protozoa, and the ITS region for fungi (Pace et al., 1986; Sogin, 1989; Schoch et al., 2012) —and enabled microbial classification typically at the genus level. The first ribosomal RNA gene-based studies of rumen microbial communities revealed that the rumen harbors far more microbial diversity than previously recognized (Whitford et al., 1998; Tajima et al., 1999). However, because the techniques available at the time were limited to analyzing relatively small numbers of sequences, these early studies largely reflected dominant taxa, and later work demonstrated that rumen microbial diversity extended well beyond these initial observations.

The adoption of next-generation sequencing (NGS) technologies in the mid-2000s marked a major turning point in the studies of the rumen microbial ecosystem. Whereas earlier sequencing methods could read only a single DNA fragment at a time, NGS platforms can sequence millions of fragments simultaneously, enabling rapid and much deeper characterization of entire microbial communities. As these technologies became available, they expanded the ability to investigate the rumen microbial ecosystem and generated new insights into its structure and dynamics, primarily through studies conducted in conventionally raised animals. For example, studies have suggested that the mature rumen microbial ecosystem tends to be relatively stable and resistant to change (Weimer et al., 2010b); that microbial colonization begins shortly after birth and may follow host-associated developmental trajectories toward a stable adult community (Jami et al., 2013); that microbial community composition can vary with diet and host species (Henderson et al., 2015); that host genetics may contribute to community structure (Li et al., 2019); that certain microbial taxa appear to be shared across individuals and environments (Wallace et al., 2019); and that early-arriving microbes seems to influence later community assembly through priority effects with potentially lasting impacts on rumen microbial composition (Furman et al., 2020).

As sequencing technologies continued to advance, NGS approaches expanded beyond marker-gene surveys to include whole-genome sequencing, which enabled researchers to decode the genomes of cultured microbes, and shotgun metagenomic sequencing, which allowed the sequencing of all DNA present in a sample. By directly identifying genes and metabolic pathways encoded in microbial genomes, these approaches greatly improved our ability to characterize rumen microbial diversity and functional potential, enabling taxonomic resolution at the strain level. Notably, Seshadri et al. (2018) used whole-genome sequencing to generate a reference genome collection of 410 previously isolated rumen bacteria and archaea, providing an essential resource for accurately interpreting metagenomic datasets. Beyond cataloging diversity, genome-resolved analyses have also enabled the discovery and reconstruction of novel metabolic pathways. For example, Hackmann and colleagues used genome-based approaches to identify metabolic pathways in rumen bacteria, revealing previously unrecognized functional capabilities (Hackmann et al., 2017; Hackmann and Zhang, 2023). Shotgun metagenomics further enabled cultivation-independent profiling of rumen communities, providing insight into both taxonomic composition and functional pathways, including those belonging to microbes that were not previously cultured. More recent large-scale studies have reconstructed thousands of metagenome-assembled genomes (MAGs) (Stewart et al., 2018, 2019; Xie et al., 2021), substantially expanding the catalog of uncultured rumen microbes. Beyond DNA-based sequencing, large-scale functional “omics” approaches deepened our understanding of rumen activity by showing which genes are expressed (Shi et al., 2014), which proteins are produced (Sasson et al., 2022), and which metabolites are generated (Wang et al., 2023a). Additionally, the more recent development of single-cell analysis provided direct insights into rare, low-abundance, or difficult-to-culture taxa, as demonstrated by Jia et al. (2024).

The advent of NGS and functional “omics” has generated vast datasets that reveal how rumen microbial communities respond to different experimental conditions. These approaches have also enabled the identification of genes, proteins, and metabolites associated or correlated with key production traits such as milk yield and composition, feed efficiency, growth performance, and methane emissions (e.g., Kittelmann et al., 2014; Wallace et al., 2019; Xue et al., 2020; Sato et al., 2024; Kobel et al., 2025). Although the idea of modulating the rumen microbial community composition is not new, these findings have renewed interest in microbial manipulation as a strategy to enhance ruminant production efficiency and sustainability. However, developing effective and long-lasting modulatory strategies requires a clear understanding of the mechanisms that determine rumen microbial composition, and these mechanisms remain only partially understood (Mizrahi and Jami, 2021). Additionally, it is important to emphasize that associations and correlations, despite their value, remain observational and do not establish causality. An association indicates that two variables co-occur, while a correlation specifically quantifies the strength and direction of their relationship. Neither, however, can demonstrate cause and effect. For instance, the presence of *Helicobacter pylori* in patients with gastric ulcers was insufficient to prove it caused the disease (Marshall and Warren, 1984). Causality was only established when Barry Marshall ingested *H. pylori* himself and subsequently developed the disease (Marshall et al., 1985) —a drastic approach, but I suppose the Nobel Prize made it worthwhile! Notably, this case involved a relatively “simple system” with a single microbial species. In contrast, the rumen is an exceptionally complex ecosystem comprising thousands of interacting microbes, making causal inference far more challenging and requiring more sophisticated and controlled experimental designs. In this context, reintroducing gnotobiotic ruminant models offers a way to isolate specific factors that may shape rumen microbial composition and function and to rigorously establish causal relationships within the rumen microbial ecosystem.

## 3 Revisiting gnotobiotic ruminant models to dissect host–microbe interactions

Gnotobiotic animals provide a controlled experimental framework for investigating host–microbial relationships, thereby enabling the disentanglement of causal relationships. Germ-free animals can be generated and colonized with single microbial species, defined consortia, or complete microbial communities. Additionally, this approach allows for controlled microbial transplantation experiments, including transfers between individuals or across host species. The importance of gnotobiotic animals for studying host–microbial interactions becomes evident when considering the key limitations of current experimental approaches. In conventionally raised animals, microbial communities vary among individuals, making it impossible to establish identical baseline conditions. This variability complicates the interpretation of experimental outcomes. For example, differences in initial microbial communities can lead to variable fermentation responses, making it difficult to attribute observed effects specifically to the treatment rather than to pre-existing microbial variation. Consistent with this, evidence from human studies demonstrates that baseline gut microbial composition can determine individual responses to dietary interventions, leading to distinct responder and non-responder phenotypes (Hoffmann Sardá et al., 2025). Additionally, the large number of microbial taxa complicates efforts to identify causal links between microbes and traits of interest in both *in vivo* studies and *in vitro* mixed-culture systems. In such high-dimensional systems, association and correlation analyses inherently increase the likelihood of detecting statistically significant correlations by chance alone. For example, at a significance threshold of α = 0.05, testing 20 microbial taxa would be expected to yield approximately one false-positive association, whereas testing 500 taxa would yield approximately 25, even in the absence of true biological effects. Notably, studies typically identify only a small subset of microbes associated with traits of interest, further underscoring the difficulty of distinguishing true biological signals from spurious associations. Although *in vitro* systems for pure culture and co-culture provide greater control over microbial environments and are well-suited for studying microbe-microbe interactions, they lack essential host physiological processes—such as absorption, immune responses, rumen motility, and digesta passage—that influence microbial composition and activity *in vivo*. Therefore, gnotobiotic models represent a critical tool to overcome these limitations, enabling controlled investigation of host–microbial interactions and the identification of causal factors shaping rumen microbial composition.

The gnotobiotic approach provides a framework to investigate the multiple factors that potentially impose selective pressures on rumen microbial composition. These drivers can be broadly categorized as host-derived, environmental, and ecological, and examples of how gnotobiotic models can be applied to address specific questions related to these factors are presented below. The more direct effects of host genetics can be evaluated using gnotobiotic models by colonizing germ-free animals from different genetic backgrounds with the same defined microbial community, enabling assessment of how host genetic variation influences microbial colonization and metabolic activity while controlling for differences in initial microbial exposure. In addition to genetics, the host immune system seems to represent a key, yet largely underexplored, driver of microbial community assembly. Evidence from non-ruminant animals highlights the role of immune-mediated components—particularly antibodies such as immunoglobulin A derived from maternal milk and the developing immune system of the offspring—in regulating intestinal microbial community composition and function (Rogier et al., 2014; Nakajima et al., 2018). Although the rumen epithelium lacks the organized mucosal immune structures found in the intestine (Steele et al., 2016), the secretion of immunoglobulin A into milk and the substantial flow of saliva containing immunoglobulin A into the rumen (Mach and Pahud, 1971) create opportunities for direct interactions between antibodies and rumen microbes. Gnotobiotic models provide a powerful approach to experimentally investigating these interactions. For example, germ-free animals colonized with a defined microbial consortium and supplemented with antibodies derived from maternal milk could be used to assess how these immune factors influence microbial establishment and persistence in the rumen. Similarly, such models could be used to evaluate how key microbial taxa modulate the development of the offspring's immune response and the effects of antibody production on rumen microbial function.

With respect to environmental factors, the impact of diet composition, nutrients, or specific components like prebiotics on rumen microbial community structure and function can be evaluated using animals colonized with defined microbial consortia. In addition to diet, gnotobiotic models provide the opportunity to investigate the role of early-life microbial exposure in shaping rumen microbial assembly. For instance, germ-free neonates can be selectively exposed to microbes derived from the maternal vaginal canal, colostrum, or feed to evaluate how different sources of initial colonizers influence microbial establishment, persistence, and function. Sequential or controlled introduction of microbial groups can also be used to test priority effects, determining how early colonizing microbes influence the recruitment and activity of later-arriving species and ultimately shape the structure and function of the rumen microbial ecosystem. Another aspect that can be explored is the contribution of the host to oxygen removal from the rumen. It is often assumed that rumen anoxia is driven by facultative anaerobic microbes consuming oxygen; however, there is evidence from other species that it is often the host, rather than microbes, that removes most of the oxygen from the gastrointestinal tract (Litvak et al., 2018).

In addition, gnotobiotic models can be used to investigate ecological interactions among rumen microbes and how cooperative metabolic networks shape community assembly and function. These interactions are particularly important for processes such as fiber degradation and methane production, which remain incompletely understood. Germ-free animals can be colonized with defined microbial consortia comprising key functional groups to evaluate how specific microbial populations contribute to fiber degradation efficiency and methane production. Furthermore, the controlled inclusion or exclusion of specific microbial taxa allows assessment of their roles in key processes such as hydrogen production, interspecies hydrogen transfer, and other metabolic interactions.

Together, these examples highlight the unique potential of gnotobiotic models to disentangle complex host, environmental, and ecological drivers of rumen microbial assembly, enabling a more mechanistic understanding of causality within the rumen ecosystem and informing strategies to improve feed efficiency, animal performance, and environmental sustainability in ruminant production systems.

## 4 Future directions

While gnotobiotic ruminant models offer a powerful framework to establish causal relationships in host–microbe interactions, several practical considerations remain. Compared with usual gnotobiotic systems, such as mice, their use in ruminants involves logistical challenges related to animal size, housing requirements, and longer life cycle, which can limit throughput and increase experimental complexity. However, the development of centralized or shared gnotobiotic ruminant facilities could greatly enhance accessibility, reduce costs, and promote the standardization of experimental approaches across research groups, ultimately accelerating progress in this field.

Despite these challenges, renewed interest in microbial isolation and cultivation, together with advances in genome-resolved approaches, is increasingly supporting the development of defined microbial consortia that will enhance the success of gnotobiotic models. Improved recovery of previously uncultured or difficult-to-culture rumen microbes will be critical to better represent functional diversity and increase the ecological relevance of these systems. In parallel, the use of small ruminants, such as lambs or goat kids, provides a practical and scalable platform to investigate mechanisms that are broadly applicable to larger ruminant species, including dairy and beef cattle.

It is also important to recognize that the cost associated with gnotobiotic ruminant experiments is substantial. However, these models are uniquely suited to generate mechanistic insights that cannot be obtained from conventional *in vivo* or *in vitro* approaches. As our understanding of the factors governing microbial community assembly improves, these insights can be translated into targeted and cost-effective strategies to manipulate the rumen microbiome. Ultimately, this knowledge will enable the development of precision interventions aimed at improving feed efficiency, enhancing animal performance, and reducing the environmental impact of ruminant production systems.

## Data Availability

The original contributions presented in the study are included in the article/supplementary material, further inquiries can be directed to the corresponding author.
